# The EUCAST Disk Diffusion Method for Antimicrobial Susceptibility Testing of Oral Anaerobes

**DOI:** 10.1111/apm.70002

**Published:** 2025-02-09

**Authors:** Anne Birkeholm Jensen, Ellen Frandsen Lau, Thomas Greve, Niels Nørskov‐Lauritsen

**Affiliations:** ^1^ Department of Dentistry and Oral Health Aarhus University Aarhus Denmark; ^2^ Department of Clinical Microbiology Aarhus University Hospital Aarhus Denmark; ^3^ Department of Clinical Microbiology Odense University Hospital Odense Denmark

**Keywords:** antibiotic resistance, oral microorganisms, periodontitis

## Abstract

There is a need for standardized methods for antimicrobial susceptibility testing (AST) of anaerobic bacteria involved in oral and extra‐oral infections. We tested the recently published EUCAST disk diffusion method for rapidly growing anaerobes on selected oral anaerobes. AST of 20 strains of *Prevotella* spp., 11 strains of *Porphyromonas gingivalis, and* six *Fusobacterium nucleatum* complex strains was performed with amoxicillin and metronidazole disks using EUCAST guidelines. Plates were incubated anaerobically, and inhibition zones were evaluated after 20 h (EUCAST recommendations) and again after 44 h. The recommended agar supported the growth of all 38 strains. Twenty‐hour incubation was sufficient for the assessment of inhibition zone diameters of *Fusobacterium* strains. Although approved for *Prevotella*, an extended study of *Prevotella* species showed inconsistent growth within the EUCAST time limit of 20 h for some strains. All *P. gingivalis* strains required 44 h of incubation for the evaluation of inhibition zones. The EUCAST disk diffusion method for AST of rapidly growing anaerobes is applicable to members of the *Fusobacterium nucleatum* complex. *P. gingivalis* and several oral strains of *Prevotella* needed 44 h of incubation to enable reading of diffusion diameter. Further studies are necessary to validate the prolonged incubation of slow‐growing anaerobes.

## 1 Introduction

Anaerobic bacteria are predominant members of the oral microbiota and are commonly involved in intra‐ and extra‐oral infections that are treated with antimicrobial agents [1, 2]. Antimicrobial treatment may also be considered in severe cases of periodontitis after failure of conventional therapy [3], including severe periodontitis in children and adolescents [4]. The subgingival microbiota is polymicrobial, and empirical treatment with broad‐spectrum antimicrobial agents or combination therapy with amoxicillin (AMX) and metronidazole (MET) is widely applied [4].

MET resistance is relatively infrequent, whereas AMX resistance is more common [5, 6]. In most cases, up to 10% of subgingival isolates from the oral cavity are resistant to MET and AMX, but with a wide variance in resistance patterns within species and across geographical locations [7]. Antimicrobial susceptibility testing (AST) methodology differs considerably between studies, which makes comparisons difficult and impedes surveillance of antimicrobial resistance [8, 9, 10].

AST of anaerobic bacteria is a difficult undertaking due to the fastidious and slow‐growing nature of many anaerobes. The gold standard method is the agar dilution or microbroth dilution test, but these methods are expensive and time‐consuming compared to the disk diffusion method [11]. Hence, the dilution tests are not preferred for routine laboratory testing. Standardized methods for AST are published by the European Committee on Antimicrobial Susceptibility Testing (EUCAST).

In 2021, an EUCAST disk diffusion method for the susceptibility testing of rapidly growing anaerobes was published [12]. The study included a large number of *Bacteroides* spp. and one strain of each of the species *Prevotella melaninogenica*, *Fusobacterium necrophorum* , *Clostridium difficile*, *Clostridium perfringens* , and *Cutibacterium acnes*, and readings were performed after 16–20 h, which is a standard EUCAST criterion. The applicability of the method was confirmed for the *Bacteroides* group [13] and for *Prevotella* species [14].

Changing the methodology of the test system, or transferring breakpoints from one species to another, may result in unreliable categorizations of susceptibility or resistance [14]. We therefore wanted to test the EUCAST disk diffusion method for rapidly growing anaerobes for AST of AMX and MET on oral anaerobic strains of the *Fusobacterium nucleatum* complex, *Prevotella* spp., and *Porphyromonas gingivalis*.

## 2 Materials and Methods

### 2.1 Bacterial Strains and Culture Conditions

*Clostridium perfringens* (ATCC 13124^T^), *Bacteroides fragilis* (ATCC 25285^T^ and multiresistant BF018 (DCMOUH0018B)) served as quality control strains [13].

Twenty strains of four *Prevotella* species, 11 *P. gingivalis* strains, and six *Fusobacterium nucleatum complex strains* were investigated (Table 1). The strains were subcultured anaerobically (80% N2, 10% CO2, 10% H2) on M6 agar plates without selective agents [15] twice before being used for AST. The strains were subjected to AST after 48 h of incubation.

**TABLE 1:** Anaerobic strains subjected to AST.

Organism	Strain designation	Origin
*Prevotella melaninogenica*	ATCC 25845^T^	Sputum, USA
*Prevotella melaninogenica*	HG73	Oral clinical isolate, T.J.M van Steenbergen, the Netherlands
*Prevotella melaninogenica*	HG118	Oral clinical isolate, T.J.M van Steenbergen, the Netherlands
*Prevotella intermedia*	CCUG 24041^T^	Empyema, Germany
*Prevotella intermedia*	AH 8291‐E	Oral clinical isolate, E. Könönen, Finland
*Prevotella intermedia*	AHN 10754	Oral clinical isolate, E. Könönen, Finland
*Prevotella intermedia*	AHN 8290	Oral clinical isolate, E. Könönen, Finland
*Prevotella intermedia*	AHN 8764	Oral clinical isolate, E. Könönen, Finland
*Prevotella intermedia*	OMZ248	Oral clinical isolate, R. Gmür, Switzerland
*Prevotella intermedia*	OMZ324	Oral clinical isolate, R. Gmür, Switzerland
*Prevotella nigrescens*	CCUG 9560^T^	Gingivitis, UK
*Prevotella nigrescens*	AHN 8272	Oral clinical isolate, E. Könönen, Finland
*Prevotella nigrescens*	AHN 8292	Oral clinical isolate, E. Könönen, Finland
*Prevotella nigrescens*	OMZ251	Oral clinical isolate, R. Gmür, Switzerland
*Prevotella nigrescens*	OMZ265	Oral clinical isolate, R. Gmür, Switzerland
*Prevotella pallens*	NCTC 13042^T^	Saliva, Finland
*Prevotella pallens*	AHN 8275	Oral clinical isolate, E. Könönen, Finland
*Prevotella pallens*	AHN 8404	Oral clinical isolate, E. Könönen, Finland
*Prevotella pallens*	AHN 8431	Oral clinical isolate, E. Könönen, Finland
*Prevotella pallens*	AHN 8858	Oral clinical isolate, E. Könönen, Finland
*Porphyromonas gingivalis*	ATCC 33277^T^	Gingiva
*Porphyromonas gingivalis*	ATCC 53978	Clinical isolate
*Porphyromonas gingivalis*	EF14449	Periodontitis, G. Dahlén, Sweden
*Porphyromonas gingivalis*	HG184	Periodontitis, G. Dahlén, Sweden
*Porphyromonas gingivalis*	OMGS 946	Periodontitis, G. Dahlén, Sweden
*Porphyromonas gingivalis*	AHN 9176	Periodontitis, E. Könönen, Finland
*Porphyromonas gingivalis*	BH 6	Periodontitis, A.J. van Winkelhoff, the Netherlands
*Porphyromonas gingivalis*	OMZ 409	Periodontitis, R. Gmür, Switzerland
*Porphyromonas gingivalis*	IOOS 577	Periodontitis, S. Ali, Norway
*Porphyromonas gingivalis*	GH6	Oral clinical isolate, M. Curtis, UK
*Porphyromonas gingivalis*	PGF7 (W83)	Oral clinical isolate
*Fusobacterium nucleatum*	ATCC 25586^T^	Cervico‐facial lesion
*Fusobacterium nucleatum*	IOOS 135	Clinical isolate, Denmark
*Fusobacterium periodonticum*	ATCC 33693^T^	Periodontitis
*Fusobacterium polymorphum*	ATCC 10953^T^	Gingivitis
*Fusobacterium polymorphum*	IOOS 98	Clinical isolate, Denmark
*Fusobacterium pseudoperiodonticum*	IOOS 12	

### 2.2 Antimicrobial Susceptibility Testing

The disk diffusion method for anaerobic bacteria was carried out following EUCAST guidelines and the 15‐15‐15 rule [12, 16, 17]. Briefly, the strains were cultivated for 48 h on agar plates. Bacterial suspensions were prepared in 0.9% saline to a McFarland density of 1.0 and used within 15 min. Fastidious Anaerobe Agar with 5% horse blood (FAA‐HB) plates (Statens Serum Institute, SSI, Copenhagen, DK) were inoculated using a sterile wet cotton swab in three directions. Antibiotic disks (AMX (2 μg) and MET (5 μg)) (Oxoid/Thermo Fischer Scientific, Basingstoke, UK) were placed on separate inoculated FAA‐HB agar plates no later than 15 min after inoculation. The plates were incubated no more than 15 min after placement of the antibiotic disks. The plates were incubated anaerobically (80% N_2_, 10% CO_2_, 10% H_2_) (Whitley A35 anaerobic workstation, West Yorkshire, UK) at 35°C ± 1°C and visually inspected after 20 and 44 h. The quality and confluence of growth, the appearance of the zone edge, and the ease with which the zone diameter could be measured were evaluated according to EUCAST criteria [12, 18]. AST was performed in biological triplicates.

#### 2.2.1 Test for β‐Lactamase Production

Strains that resulted in small inhibition zone diameters around the AMX disk were tested for β‐lactamase production with the nitrocefin disk (Remel Inc., Lenexa, KS 66215, USA). Colonies were inoculated on the nitrocefin disk, incubated at room temperature for 5 min, and a shift from no color to pink indicated β‐lactamase production.

### 2.3 Data Analysis

All data analyses were performed using the SciPy program (https://docs.scipy.org/doc/scipy/index.html). The results were divided into three groups for each disk: “No growth” indicating that the strain did not show sufficient growth for reading of the inhibition zone diameter, “Inhibition zone diameter (IZD) = 6” indicating sufficient growth but no inhibition zone circumscribing the disk, and “IZD > 6” indicating sufficient growth and readable IZD (the diameter of an antibiotic disk is 6 mm). The results from the triplicated runs of each disk were plotted together for visual inspection of the reproducibility.

The range of the IZDs for each strain in the related three runs was indicated as a standard deviation (95% confidence interval) [17].

As a rule, the standard deviations were analyzed using the results from the “IZD > 6” group. If one strain presented with two readings in the “IZD > 6” group and one reading in the “No growth,” the standard deviation was calculated based on the results from the “IZD > 6” group only. If a strain presented with only one result in the “IZD > 6” or none, the data were not used for statistical analysis. The coefficient of variation of the mean was calculated as the ratio of the standard deviation to the mean for each species and antibiotic disk after 20 and 44 h [19], and was used to compare the reproducibility of the method after 20 and 44 h.

### 2.4 Whole Genome Sequencing

*Fusobacterium* strains IOOS 12, IOOS 98, and IOOS 135 were genome sequenced using Oxford Nanopore Technology as previously described [20]. The nucleotide sequences are deposited at DDBJ/ENA/GenBank under accession JAZAPL000000000, CP144257, and CP144258, respectively.

## 3 Results

In all runs, the reference strains resulted in IZDs within the recommended target range [21] (data not shown).

Overall, both reference and test strains attained confluent growth on FAA‐HB plates, although different incubation times were needed. We observed a trend of increased IZDs and variation between triplicates when incubation was prolonged from 20 to 44 h.

The distribution of IZDs was symmetric, but with overall large IZDs (Figures S1–S3). It was only possible to estimate the difference between wild type (no phenotypically detectable resistance mechanisms) and non‐wild‐type isolates for *Prevotella* species, because several resistant strains were present in this population (Figure S1).

### 3.1 Prevotella Species

**FIGURE 1:**
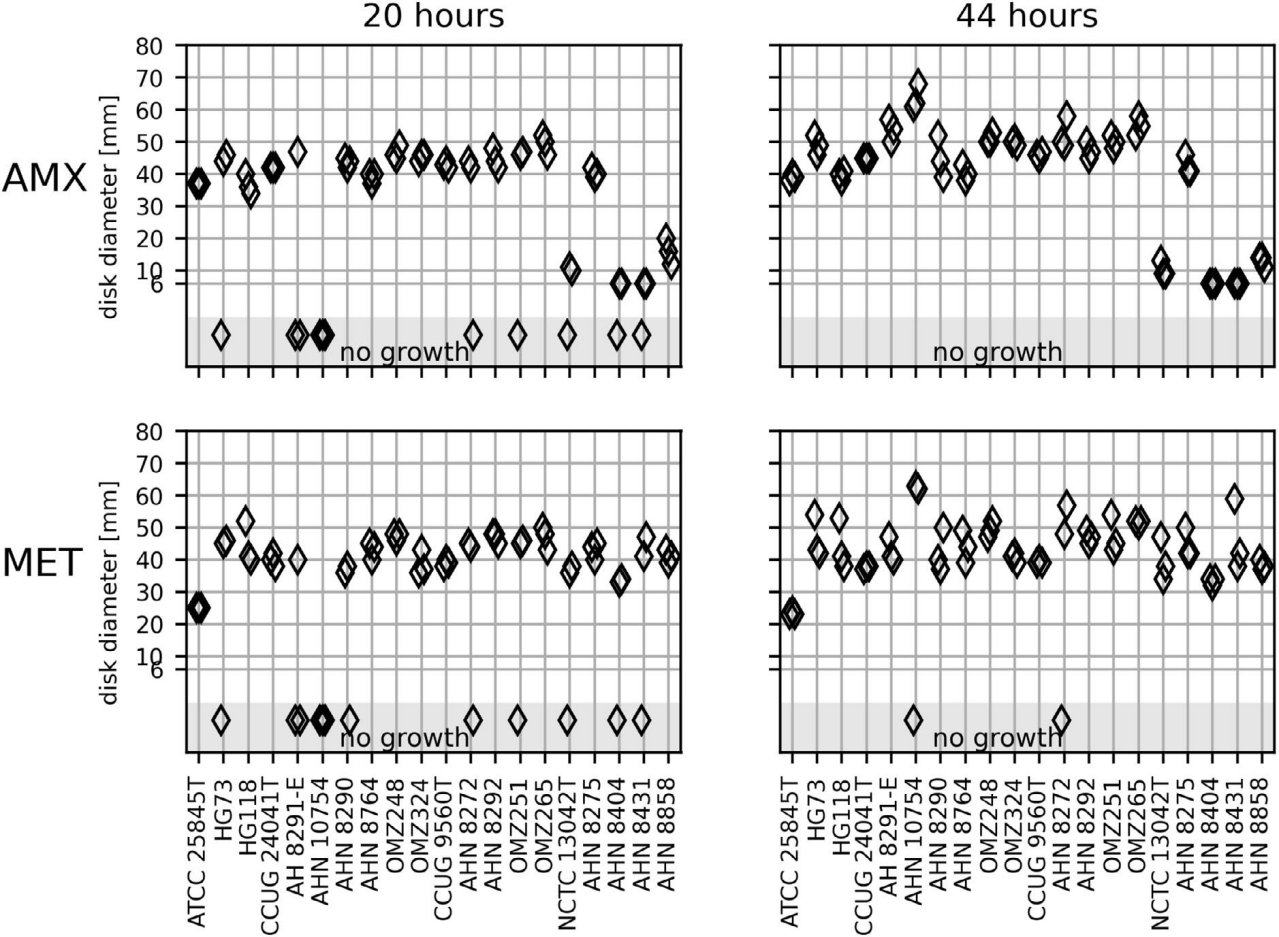
The inhibition zone diameters in mm (*y*‐axis) obtained on FAA‐HB and twenty *Prevotella* (*x*‐axis) on amoxicillin (AMX) and metronidazole (MET) disks after 20 and 44 h of incubation. Triplicated runs. Six millimeters is the diameter of the antibiotic disk and indicates no inhibition zone around the antibiotic disks. The light gray “no growth” shows the strains that did not grow in one or more runs.

The 20 *Prevotella* strains of four species showed confluent growth on FAA‐HB at different incubation times, but IZDs varied between triplicates and between readings after 20 and 44 h (Figure 1). After 20 h, 12 strains showed sufficient growth in all triplicates for measurement of IZDs around the AMX disk, and 11 strains around the MET disk. For both disks, no growth was recorded for six strains in one of the triplicates, one strain in two of the triplicates, and one strain in all the triplicates. After 44 h, all *Prevotella* strains showed confluent growth and readable IZDs around AMX in all triplicates, except for two strains that showed no growth in one of the triplicates on the MET disk after 44 h (Figure 1).

The pairwise comparison of the IZDs based on the measurable zone of inhibition after 20 and 44 h of incubation indicated that the AMX zones were consistent across 14 of the 20 strains. The MET zones were consistent across nine strains (Figure 1). The best reproducibility was obtained after 20 h on both disks based on the coefficients of variance of the mean (Table 2 and Figure S4). However, it should be noted that the 20‐h results for the AMX disk were restricted to two measurements for some of the strains. Table S1 shows the percentages of the readings resulting in sufficient growth for interpretation of IZDs after 20 and 44 h.

**TABLE 2:** The coefficient of variance of the mean.

Species	AMX		MET	
	20 h	44 h	20 h	44 h
*Prevotella*	0.65	0.98	0.69	1.14
*Porphyromonas*	—	0.57	—	0.44
*Fusobacterium*	0.23	0.34	0.28	0.27

Four *Prevotella* strains were positive for β‐lactamase production (AHN 8404, AHN 8431, AHN 8858 and NCTC 13042^T^, all *Prevotella pallens* ). Interpretation based on inferred susceptibility breakpoints from ampicillin (zone diameter breakpoint ≤ 25 mm corresponds to resistant) showed a correlation between β‐lactamase production and a zone diameter breakpoint of < 25 mm. Finally, the modest IZDs around the MET of *P. melaninogenica* ATCC 25845^T^ (mean 25 mm after 20 h and 23 mm after 44 h) contrasted with the large zones observed by all other *Prevotella* strains. However, ATCC 25845^T^ shall be characterized as susceptible according to EUCAST guidelines approved for *Prevotella* spp. (MET zone diameter breakpoint for *Prevotella* is 22 mm).

### 3.2 *P. gingivalis*

**FIGURE 2:**
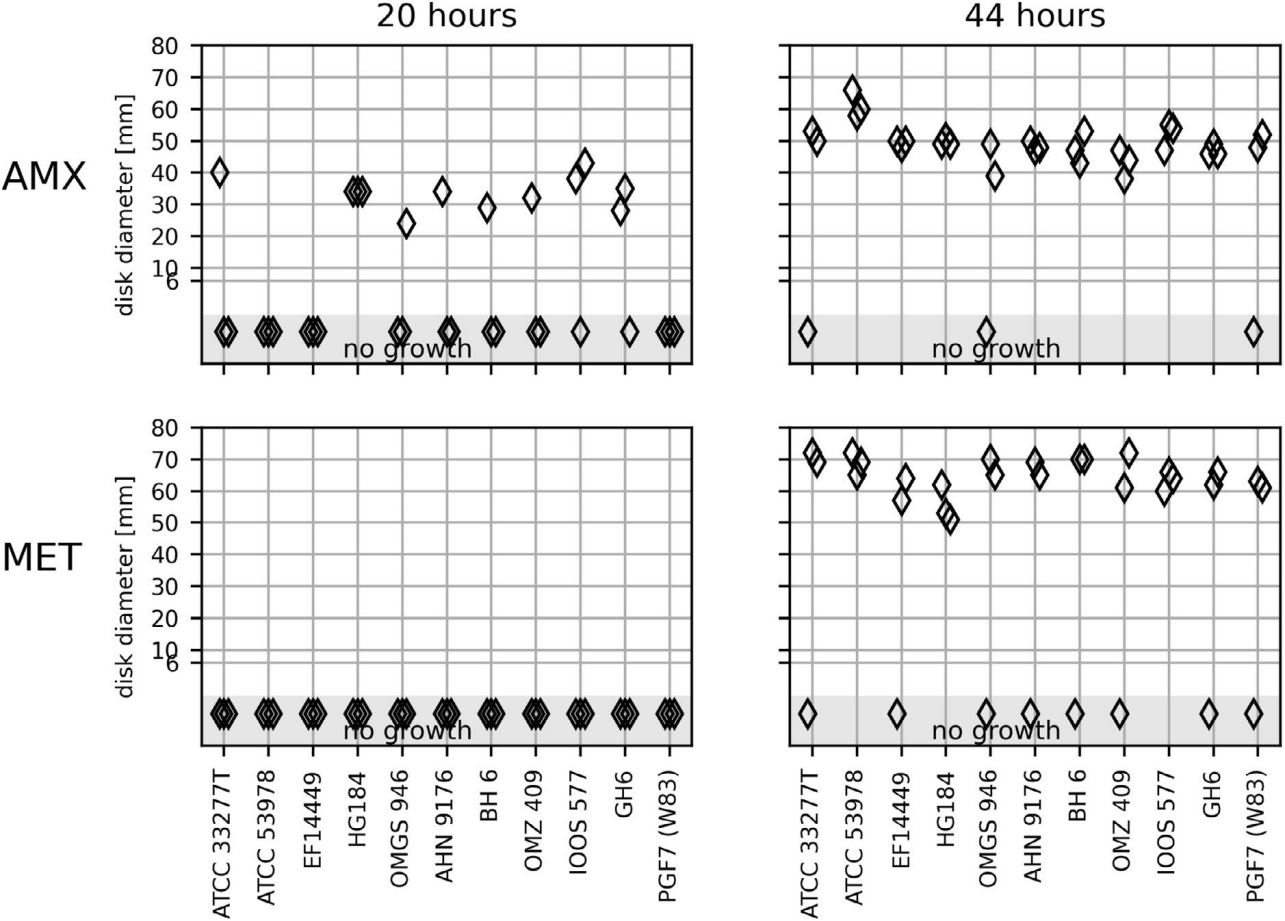
The inhibition zone diameters in mm (*y*‐axis) obtained on FAA‐HB and eleven *P. gingivalis* (*x*‐axis) on amoxicillin (AMX) and metronidazole (MET) disks after 20 and 44 h of incubation. Triplicated runs. Six millimeters is the diameter of the antibiotic disk and indicates no inhibition zone around the antibiotic disks. The light gray “no growth” shows the strains that did not grow in one or more runs.

Only one strain gave rise to readable IZDs in triplicate after 20 h, and only for the AMX disk (Figure 2). All 11 strains of *P. gingivalis* showed confluent growth on FAA‐HB after 44 h incubation in at least two of the triplicates (Figure 2).

The IZDs on the MET disk were of considerable size (50–70 mm), almost reaching the diameters of the agar plates. The small rim of growth in the periphery may have impaired the reading of zones. It is possible that a similar proportion of strains expressed growth around AMX and MET disks after 20 h, but that the growth in the periphery of the MET plates escaped detection (Figure 2). The inhibition zones after 44 h of incubation varied considerably for most strains with the MET disk and for five strains with the AMX disk (Figure S5).

There are no EUCAST zone diameter breakpoints for *Porphyromonas* spp. Although only readable after 44 h, the mean IZDs larger than 30 mm could indicate susceptibility to both AMX and MET.

### 3.3 *F. nucleatum* Complex

**FIGURE 3:**
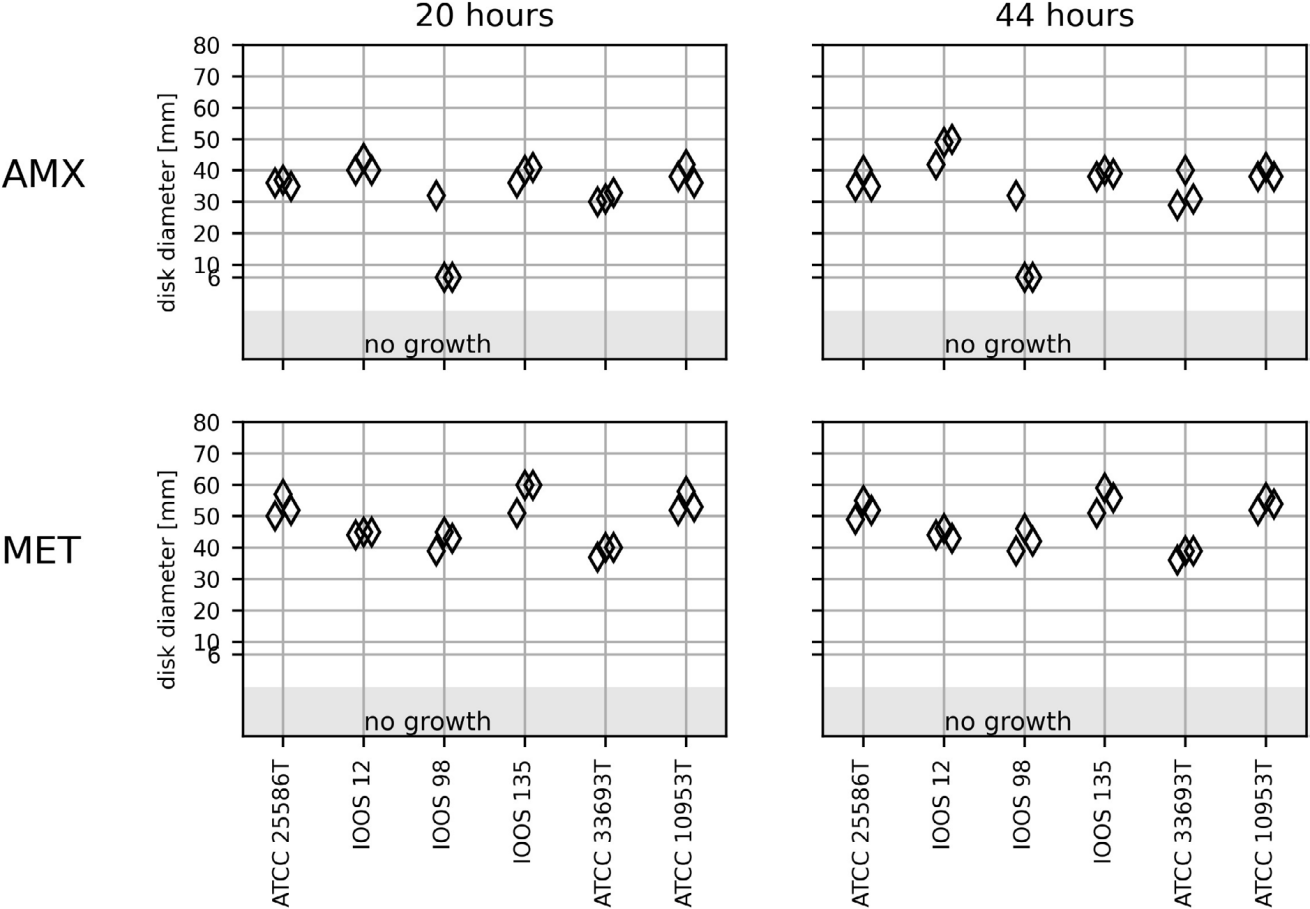
The inhibition zone diameters in mm (*y*‐axis) obtained on FAA‐HB for the six *Fusobacterium* (*x*‐axis) on amoxicillin (AMX) and metronidazole (MET) disks after 20 and 44 h of incubation. Triplicated runs. Six millimeters is the diameter of the antibiotic disk and indicates no inhibition zone around the antibiotic disks. The light gray area “no growth” shows the strains that did not grow in one or more runs.

Genus *Fusobacterium* encompasses a heterogeneous group of species, and only *F. necrophorum* is approved for EUCAST AST by disk diffusion [16]. The three clinical *Fusobacterium* strains were genome sequence identified to species level and listed with species designation in Table 1. The four *Fusobacterium* species are closely related to *F. nucleatum* . *Fusobacterium polymorphum* and *Fusobacterium pseudoperiodonticum* constitute genuine species by genome analysis [22, 23], but have not yet been validly published. The six *Fusobacterium* strains showed confluent growth and readable IZDs on FAA‐HB in triplicates after 20 h. *F. pseudoperiodonticum* IOOS 12 and *Fusobacterium periodonticum* ATCC 33693^T^ varied more than 5 mm in IZDs of the AMX disk after 44 h (Figure 3). *F. polymorphum* IOOS 98 had an inhibition zone of approximately 30 mm around the AMX disk in one of the triplicates compared to no zone in the remaining measurements (Figure 3). This strain tested positive for β‐lactamase activity and the presence of a complete class D β‐lactamase OXA‐85 gene, which was originally described in this species [24]. The inhibition zone of 30 mm was concluded to be a random biological error and excluded from further analysis. The coefficient of variance of the mean was similar after 20‐ and 44‐h incubation for both AMX and MET (Table 2 and Figure S6).

No EUCAST zone diameter breakpoints are available for *F. nucleatum* . The susceptibility interpretation can be inferred from ampicillin. The zone diameter breakpoint for *F. necrophorum* and ampicillin is 27 mm. All *Fusobacterium* were considered susceptible except the ß‐lactamase‐positive *F. polymorphum* IOOS 98. The EUCAST zone diameter breakpoint for *F. necrophorum* and MET is 30 mm, and all the strains may be considered susceptible to MTX.

## 4 Discussion

The present study applied the EUCAST disk diffusion method for rapidly growing anaerobes to AST of oral, anaerobic strains of *Prevotella* spp., *P. gingivalis*, and the *F. nucleatum* complex. The lack of or poor growth after 20 h for some strains of *Prevotella* and *P. gingivalis* did not spring from large IZDs because the plates were clearly readable after 44 h; however, a small rim of growth in the periphery could be a challenge for the judgment of zones. Recently, EUCAST has tried to overcome the challenge with AST of anaerobes by modifying the disk diffusion method for rapidly growing anaerobes [14]. Due to the lack of readable zones after 20 h incubation of agar plates and the comprehensiveness of the agar dilution, antibiotic treatment of anaerobes is often based on empirical evidence rather than an antibiotic susceptibility profile [25].

The EUCAST disk diffusion for rapidly growing anaerobes was developed using, e.g., *F. necrophorum*. In our study, all six strains of four oral *Fusobacterium* species resulted in IZDs that could be read after 20 h on FAA‐HB, and detection of AMX resistance was in accordance with the production of β‐lactamase. Previously reported extension to 48 h incubation [9, 10] does seem necessary. The small strain collection of *Fusobacterium* is a limitation of our study, and the results may be considered preliminary. Our findings support further testing of the EUCAST disk diffusion method for AST of the *F. nucleatum* complex across laboratories and geographic borders.

EUCAST clearly states that prolonged incubation of more than 20 h cannot be implemented for the disk diffusion method because IZDs read after 20 and 44 h may vary considerably [14, 16].

*P. melaninogenica* was included in the development study of EUCAST AST for rapidly growing anaerobes. We used three strains of this species, including the type strains, plus additional strains of *Prevotella intermedia* , *Prevotella nigrescens* , and *P. pallens* . In our study, several strains from each of the four species could not be read in one out of three triplicates after 20 h due to poor growth. A compliance deficit of 18% is severe, but it does not renounce the use of the test. Most AST of *Prevotella* will be successful, and preliminary guidance on treatment can be resolved after prolonged incubation of challenging strains. Reports of multidrug‐resistant *Prevotella* strains, in addition to increasing levels of resistance in both oral and extra‐oral species of *Prevotella*, indicate that resistant isolates may be encountered in the clinical setting, and such isolates should be subjected to reference AST of submitted reference laboratories [2, 26, 27].

*Porphyromonas* species are not approved for EUCAST AST and constitute a difficult undertaking. We examined 11 strains of *P. gingivalis* , and successful assessment after 20 h was the exception. Although restricted to one species, our data do not suggest *Porphyromonas* as a candidate for standard EUCAST AST. It is possible that EUCAST‐like AST with prolonged incubation could be feasible and helpful, and EUCAST accepts prolonged incubation of selected bacteria, for example, *Campylobacter jejuni* [28]. The pathogenic nature of *P. gingivalis* , in addition to the reported antimicrobial resistance across geographic borders, supports the development of a method to enable the continuous monitoring of the development of antibiotic resistance [8, 29, 30, 31]. The large IZDs for *P. gingivalis* could indicate that disk diffusion is not a suitable method for AST of *P. gingivalis* . Until a thorough understanding has been attained, microbroth minimal inhibitory concentration may be indispensable for the assessment of susceptibility of this species.

The methodological variations in studies on antimicrobial susceptibility in oral strains emphasize the need for the development of a standardized approach to AST of oral anaerobes needing prolonged incubation [8, 9, 32, 33].

The relatively small strain collection in the present study is a limitation, and the results should be interpreted accordingly. Our study would additionally have benefitted from a wider range of antimicrobials and MIC determinations of the study strains.

Our results have shown that the EUCAST disk diffusion method for rapidly growing anaerobes may be suitable for a range of orally occurring *Fusobacterium* and some *Prevotella* isolates. *P. gingivalis* is an unlikely candidate for the EUCAST disk diffusion method due to the need for prolonged incubation. Until further research is done, resistance in *P. gingivalis* should preferably be categorized by MIC determination.

## Conflicts of Interest

The authors declare no conflicts of interest.

## Supporting information


**Figure S1.** Inhibition zone diameter distributions for the 20 *Prevotella* species (60 correlates) with the amoxicillin disk (2 μg) and the metronidazole disk (5 μg) on fastidious anaerobe agar with horse blood (FAA) medium after 44 h anaerobic incubation.

**Figure S2.** Inhibition zone diameter distributions for the 11 *Porphyromonas gingivalis* (24 correlates) with the amoxicillin disk (2 μg) and the metronidazole disk (5 μg) on fastidious anaerobe agar with horse blood (FAA medium) after 44 h anaerobic incubation.

**Figure S3.** Inhibition zone diameter distributions for the six *Fusobacterium* (18 correlates) species with the amoxicillin disk (2 μg) and the metronidazole disk (5 μg) on fastidious anaerobe agar with horse blood (FAA) medium after 20 h anaerobic incubation.

**Figure S4.** Standard deviation coefficient of variance of the mean (MCV) of the inhibition zone diameters of the amoxicillin (AMX) and the metronidazol (MET) disk after 20 and 44 h on *Prevotella*. The isolates are represented on the x‐axis and the inhibition zone diameter (mm) on the *y*‐axis.

**Figure S5.** Standard deviation of the inhibition zone diameters coefficient of variance of the mean (MCV) of the amoxicillin (AMX) and the metronidazol (MET) disk after 20 and 44 h on *P. gingivalis* . The isolates are represented on the x‐axis and the inhibition zone diameter (mm) on the *y*‐axis.

**Figure S6.** Standard deviation and coefficient of variance of the mean (MCV) of the inhibition zone diameters of the amoxicillin (AMX) and the metronidazol (MET) disk after 20 and 44 h on *Fusobacterium*. The isolates are represented on the x‐axis and the inhibition zone diameter (mm) on the *y*‐axis.

**Table S1.** The number of readings resulting in sufficient growth for interpretation of inhibition zone diameters for 20 *Prevotella* (60 correlates), 11 *Porhyromonas* strains (33 correlates), and six *Fusobacterium* (18 correlates).

## Data Availability

The data that support the findings of this study are available from the corresponding author upon reasonable request.

## References

[1] F. E. Dewhirst , T. Chen , J. Izard , et al., “The Human Oral Microbiome,” Journal of Bacteriology 192, no. 19 (2010): 5002–5017, 10.1128/jb.00542-10. 20656903 PMC2944498

[2] J. Ligero‐López , E. Rubio‐Mora , M. D. Ruiz‐Bastián , M. I. Quiles‐Melero , J. Cacho‐Calvo , and E. Cendejas‐Bueno , “Antimicrobial Susceptibility Testing of Anaerobic Bacteria Causing Bacteremia: A 13‐Year (2010‐2022) Retrospective Study in a Tertiary Hospital,” Anaerobe 84 (2023): 102803, 10.1016/j.anaerobe.2023.102803. 37984560

[3] M. Sanz , D. Herrera , M. Kebschull , et al., “Treatment of Stage I‐III Periodontitis‐The EFP S3 Level Clinical Practice Guideline,” Journal of Clinical Periodontology 47, no. Suppl 22 (2020): 4–60, 10.1111/jcpe.13290. 32383274 PMC7891343

[4] W. Teughels , M. Feres , V. Oud , C. Martin , P. Matesanz , and D. Herrera , “Adjunctive Effect of Systemic Antimicrobials in Periodontitis Therapy: A Systematic Review and Meta‐Analysis,” Journal of Clinical Periodontology 47, no. Suppl 22 (2020): 257–281, 10.1111/jcpe.13264. 31994207

[5] C. Alauzet , A. Lozniewski , and H. Marchandin , “Metronidazole Resistance and Nim Genes in Anaerobes: A Review,” Anaerobe 55 (2019): 40–53, 10.1016/j.anaerobe.2018.10.004. 30316817

[6] R. J. Worthington and C. Melander , “Overcoming Resistance to β‐Lactam Antibiotics,” Journal of Organic Chemistry 78, no. 9 (2013): 4207–4213, 10.1021/jo400236f. 23530949 PMC3644377

[7] E. Ng , J. R. H. Tay , S. K. Boey , M. L. Laine , S. Ivanovski , and C. J. Seneviratne , “Antibiotic Resistance in the Microbiota of Periodontitis Patients: An Update of Current Findings,” Critical Reviews in Microbiology 50, no. 3 (2023): 1–12, 10.1080/1040841x.2023.2197481. 37140235

[8] G. Conrads , T. Klomp , D. Deng , J. S. Wenzler , A. Braun , and M. M. H. Abdelbary , “The Antimicrobial Susceptibility of *Porphyromonas Gingivalis*: Genetic Repertoire, Global Phenotype, and Review of the Literature,” Antibiotics 10, no. 12 (2021): 1438, 10.3390/antibiotics10121438. 34943650 PMC8698109

[9] K. Jepsen , W. Falk , F. Brune , R. Fimmers , S. Jepsen , and I. Bekeredjian‐Ding , “Prevalence and Antibiotic Susceptibility Trends of Periodontal Pathogens in the Subgingival Microbiota of German Periodontitis Patients: A Retrospective Surveillance Study,” Journal of Clinical Periodontology 48, no. 9 (2021): 1216–1227, 10.1111/jcpe.13468. 33934384

[10] A. Mosca , L. Miragliotta , M. A. Iodice , A. Abbinante , and G. Miragliotta , “Antimicrobial Profiles of *Prevotella* spp. and *Fusobacterium nucleatum* Isolated From Periodontal Infections in a Selected Area of Southern Italy,” International Journal of Antimicrobial Agents 30, no. 6 (2007): 521–524, 10.1016/j.ijantimicag.2007.07.022. 17954025

[11] A. Sood , P. Ray , and A. Angrup , “Antimicrobial Susceptibility Testing of Anaerobic Bacteria: In Routine and Research,” Anaerobe 75 (2022): 102559, 10.1016/j.anaerobe.2022.102559. 35417767

[12] H. Bavelaar , U. S. Justesen , T. E. Morris , et al., “Development of a EUCAST Disk Diffusion Method for the Susceptibility Testing of Rapidly Growing Anaerobic Bacteria Using Fastidious Anaerobe Agar (FAA): A Development Study Using Bacteroides Species,” Clinical Microbiology and Infection 27, no. 11 (2021): 1691–1695, 10.1016/j.cmi.2021.03.028. 33813129

[13] T. T. Stubhaug , C. G. Giske , U. S. Justesen , et al., “Antimicrobial Susceptibility Testing of Bacteroides Species by Disk Diffusion: The NordicAST Bacteroides Study,” Anaerobe 81 (2023): 102743, 10.1016/j.anaerobe.2023.102743. 37253399

[14] E. Matuschek , S. Copsey‐Mawer , S. Petersson , J. Åhman , T. E. Morris , and G. Kahlmeter , “The European Committee on Antimicrobial Susceptibility Testing Disc Diffusion Susceptibility Testing Method for Frequently Isolated Anaerobic Bacteria,” Clinical Microbiology and Infection 29 (2023): 795.e1–795.e7, 10.1016/j.cmi.2023.01.027. 36746258

[15] D. E. Hunt , J. V. Jones , and V. R. Dowell, Jr. , “Selective Medium for the Isolation of *Bacteroides gingivalis* ,” Journal of Clinical Microbiology 23, no. 3 (1986): 441–445, 10.1128/jcm.23.3.441-445.1986. 3958141 PMC268670

[16] EUCAST, The European Committee on Antimicrobial Susceptibility Testing , “EUCAST Disk Diffusion Methodology for Selected Rapidly Growing Anerobic Bacteria on Fastidious Anaerobe Agar With 5% Horse Blood (FAA‐HB),” (2023).

[17] E. Matuschek , D. F. Brown , and G. Kahlmeter , “Development of the EUCAST Disk Diffusion Antimicrobial Susceptibility Testing Method and Its Implementation in Routine Microbiology Laboratories,” Clinical Microbiology and Infection 20, no. 4 (2014): O255–O266, 10.1111/1469-0691.12373. 24131428

[18] EUCAST, The European Committee on Antimicrobial Susceptibility Testing , “Reading Guide: EUCAST Disk Diffusion for Selectd Rapidly Growing Anaerobic Bacteria on Fastidious Anaerobes Agar With 5% Horse Blood (FAA‐HB),” (2023).

[19] G. F. Reed , F. Lynn , and B. D. Meade , “Use of Coefficient of Variation in Assessing Variability of Quantitative Assays,” Clinical and Diagnostic Laboratory Immunology 9, no. 6 (2002): 1235–1239, 10.1128/cdli.9.6.1235-1239.2002. 12414755 PMC130103

[20] N. Nørskov‐Lauritsen , R. Mohey , D. S. Hansen , et al., “Genome Characterisation of Invasive *Haemophilus influenzae* in Pregnancy: The Noticeable Placental Tissue Tropism Is Distributed Across the Species Rather Than Linked With Capsulation or Particular Clones,” Pathogens 12, no. 11 (2023): 1345, 10.3390/pathogens12111345. 38003810 PMC10675716

[21] EUCAST, The European Committee on Antimicrobial Susceptibility Testing , “Routine and Extended Internal Quality Control for MIC Determination and Disk Diffusion as Recommended by EUCAST,” (2023).

[22] J. K. Kook , S. N. Park , Y. K. Lim , et al., “Genome‐Based Reclassification of *Fusobacterium nucleatum* Subspecies at the Species Level,” Current Microbiology 74, no. 10 (2017): 1137–1147, 10.1007/s00284-017-1296-9. 28687946

[23] S. N. Park , Y. K. Lim , J. H. Shin , et al., “Fusobacterium *Pseudoperiodonticum* sp. nov., Isolated From the Human Oral Cavity,” Current Microbiology 76, no. 6 (2019): 659–665, 10.1007/s00284-019-01675-y. 30937514

[24] C. Voha , J. D. Docquier , G. M. Rossolini , and T. Fosse , “Genetic and Biochemical Characterization of FUS‐1 (OXA‐85), a Narrow‐Spectrum Class D Beta‐Lactamase From *Fusobacterium nucleatum* subsp. *polymorphum* ,” Antimicrobial Agents and Chemotherapy 50, no. 8 (2006): 2673–2679, 10.1128/aac.00058-06. 16870757 PMC1538689

[25] A. N. Schuetz , “Antimicrobial Resistance and Susceptibility Testing of Anaerobic Bacteria,” Clinical Infectious Diseases 59, no. 5 (2014): 698–705, 10.1093/cid/ciu395. 24867792

[26] M. Shariff and E. Ramengmawi , “Antimicrobial Resistance Pattern of Anaerobic Bacteria Causing Lower Respiratory Tract Infections,” BioMed Research International 23, no. 1 (2023): 301, 10.1186/s12866-023-03059-6. PMC10591390 37872502

[27] N. Ulger Toprak , A. C. M. Veloo , E. Urban , et al., “A Multicenter Survey of Antimicrobial Susceptibility of Prevotella Species as Determined by Etest Methodology,” Anaerobe 52 (2018): 9–15, 10.1016/j.anaerobe.2018.05.005. 29860038

[28] EUCAST, The European Committee on Antimicrobial Susceptibility Testing , “Breakpoint Tables for Interpretation of MICs and Zone Diameters,” (2023).

[29] G. Hajishengallis , R. J. Lamont , and H. Koo , “Oral Polymicrobial Communities: Assembly, Function, and Impact on Diseases,” Cell Host & Microbe 31, no. 4 (2023): 528–538, 10.1016/j.chom.2023.02.009. 36933557 PMC10101935

[30] E. M. Kulik , T. Thurnheer , L. Karygianni , C. Walter , A. Sculean , and S. Eick , “Antibiotic Susceptibility Patterns of Aggregatibacter Actinomycetemcomitans and *Porphyromonas gingivalis* Strains From Different Decades,” Antibiotics 8, no. 4 (2019): 253, 10.3390/antibiotics8040253. 31817588 PMC6963212

[31] T. E. Rams , J. D. Sautter , and A. J. van Winkelhoff , “Emergence of Antibiotic‐Resistant *Porphyromonas gingivalis* in United States Periodontitis Patients,” Antibiotics 12, no. 11 (2023): 1584, 10.3390/antibiotics12111584. 37998786 PMC10668829

[32] D. Manoil , A. Parga , N. Bostanci , and G. N. Belibasakis , “Microbial Diagnostics in Periodontal Diseases,” Periodontology 2000 95, no. 1 (2024): 176–193, 10.1111/prd.12571. 38797888

[33] A. C. M. Veloo , W. H. Baas , F. J. Haan , J. Coco , and J. W. Rossen , “Prevalence of Antimicrobial Resistance Genes in *Bacteroides* spp. and *Prevotella* spp. Dutch Clinical Isolates,” Clinical Microbiology and Infection 25, no. 9 (2019): 1156.e9–1159.e13, 10.1016/j.cmi.2019.02.017. 30802650

