# Supplementary figures and images for: The EUCAST Disk Diffusion Method for Antimicrobial Susceptibility Testing of Oral Anaerobes
Source: APMIS. 2025 Feb 9;133(2):e70002. doi: 10.1111/apm.70002 (PMC11807598; doi:10.1111/apm.70002)

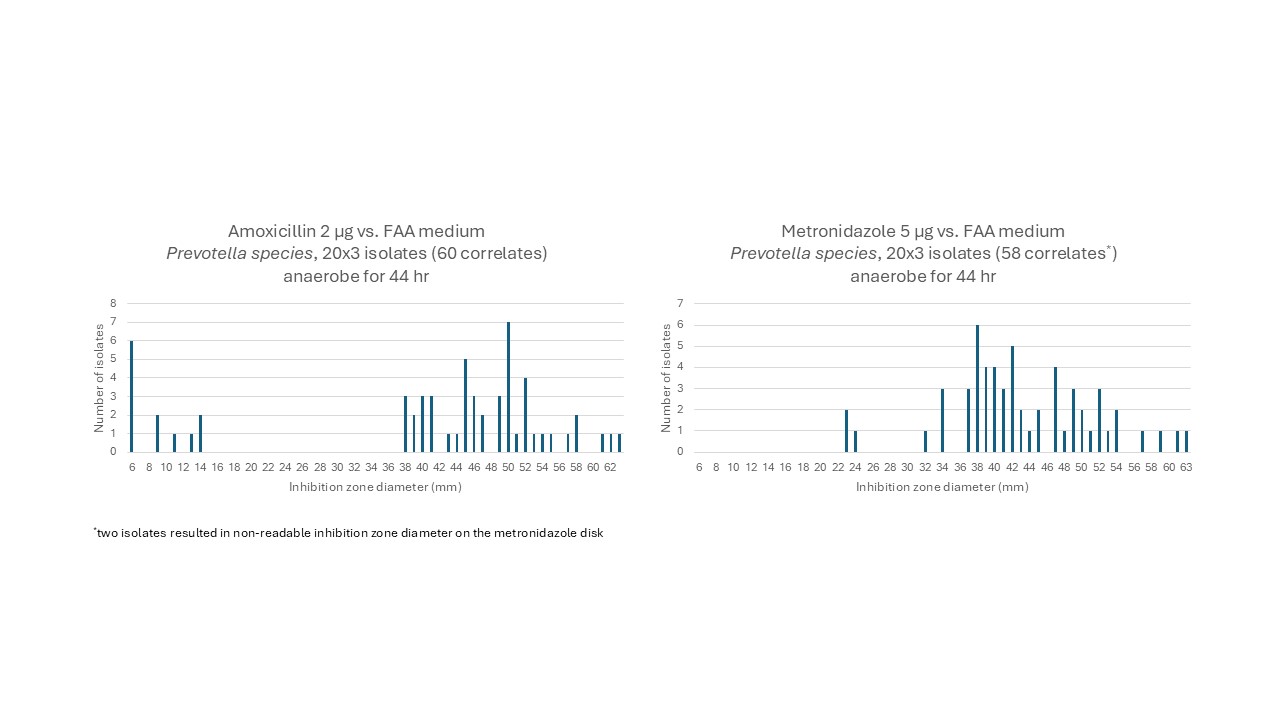

Supplement: Supplementary file 1 — Figure S1. Inhibition zone diameter distributions for the 20 Prevotella species (60 correlates) with the amoxicillin disk (2 μg) and the metronidazole disk (5 μg) on fastidious anaerobe agar with horse blood (FAA) medium after 44 h anaerobic incubation. [file APM-133-0-s002.jpg]

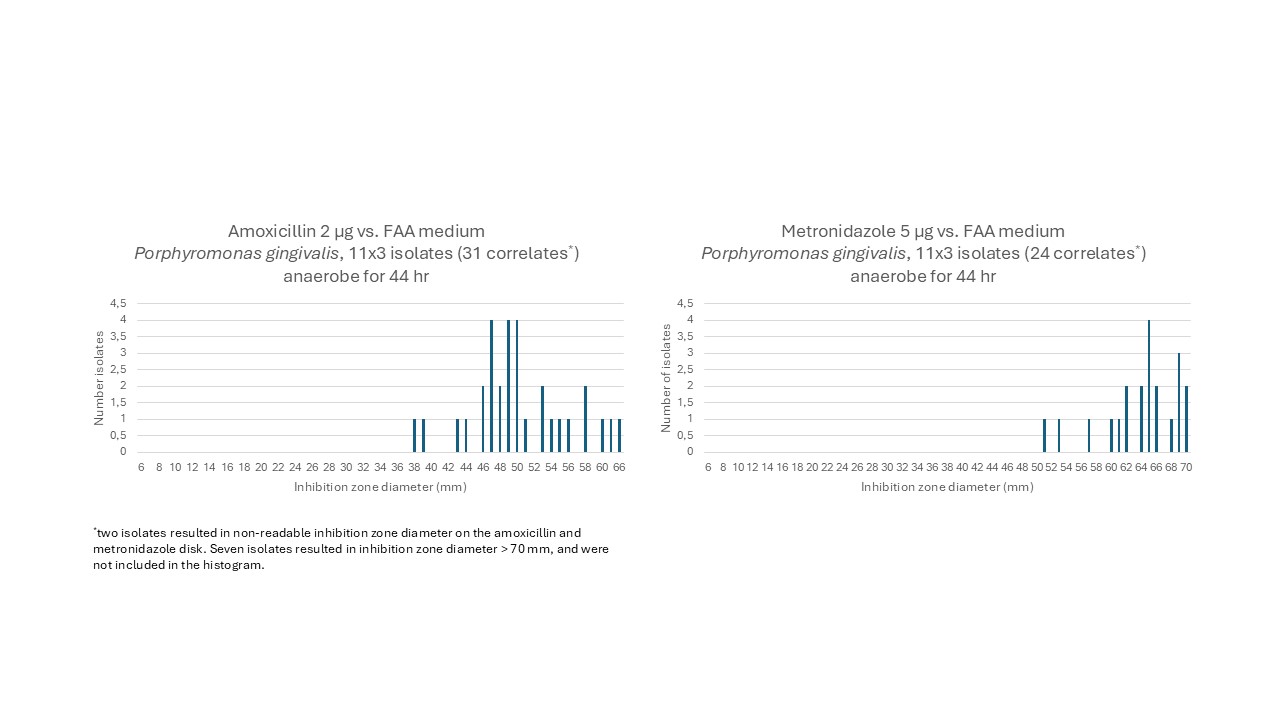

Supplement: Supplementary file 2 — Figure S2. Inhibition zone diameter distributions for the 11 Porphyromonas gingivalis (24 correlates) with the amoxicillin disk (2 μg) and the metronidazole disk (5 μg) on fastidious anaerobe agar with horse blood (FAA medium) after 44 h anaerobic incubation. [file APM-133-0-s001.jpg]

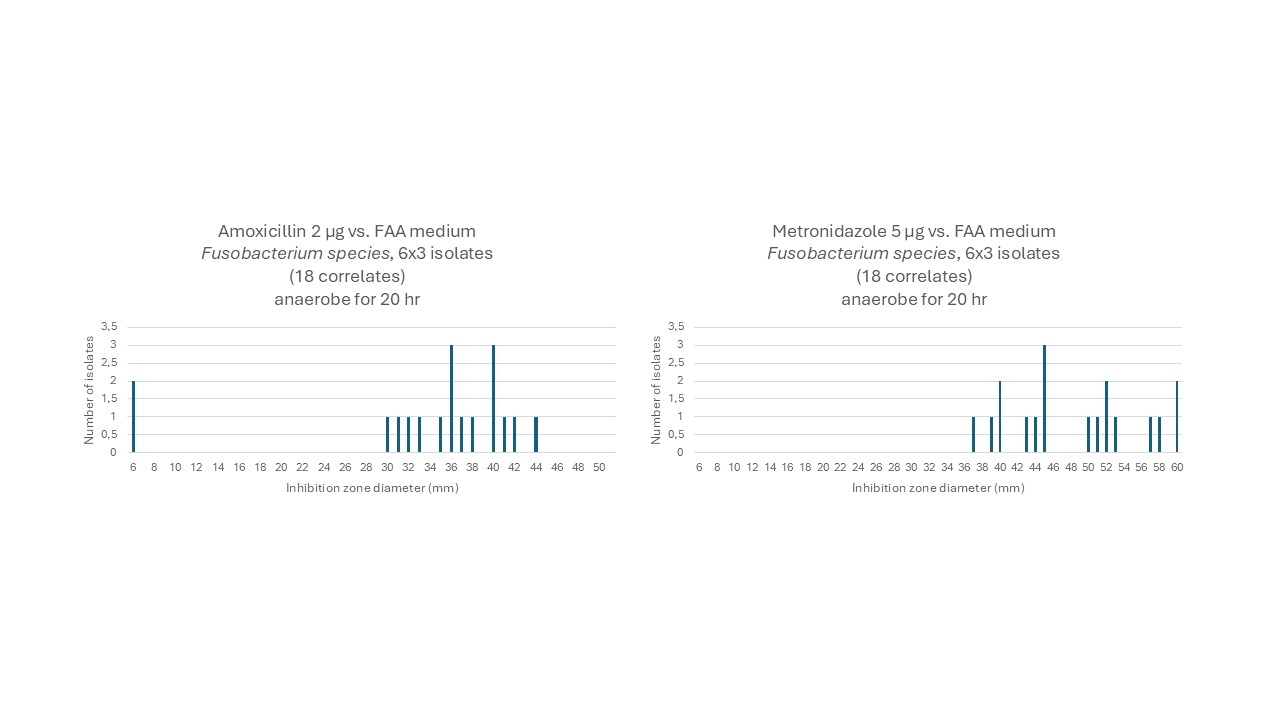

Supplement: Supplementary file 3 — Figure S3. Inhibition zone diameter distributions for the six Fusobacterium (18 correlates) species with the amoxicillin disk (2 μg) and the metronidazole disk (5 μg) on fastidious anaerobe agar with horse blood (FAA) medium after 20 h anaerobic incubation. [file APM-133-0-s007.jpg]

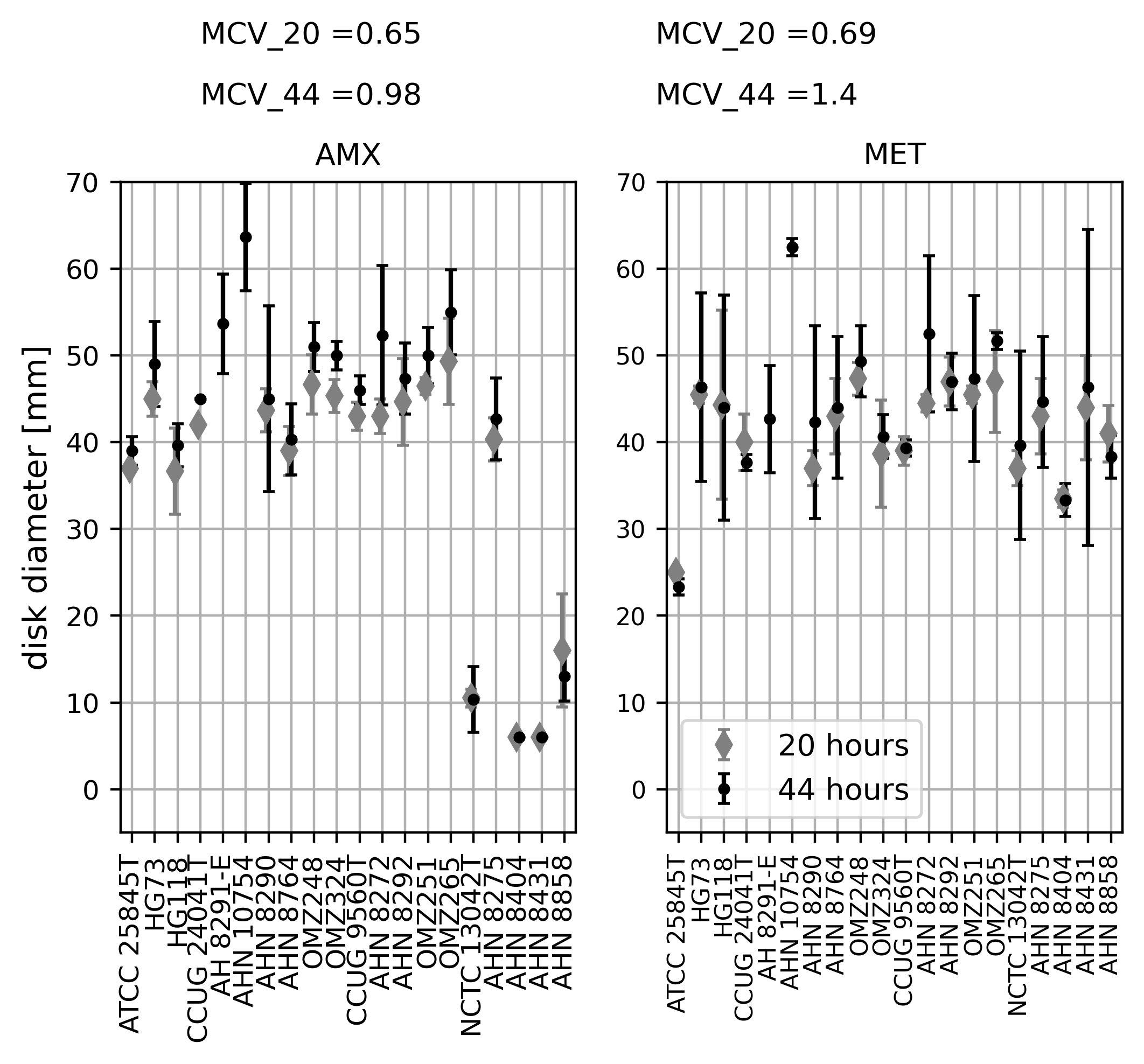

Supplement: Supplementary file 4 — Figure S4. Standard deviation coefficient of variance of the mean (MCV) of the inhibition zone diameters of the amoxicillin (AMX) and the metronidazol (MET) disk after 20 and 44 h on Prevotella. The isolates are represented on the x‐axis and the inhibition zone diameter (mm) on the y‐axis. [file APM-133-0-s003.png]

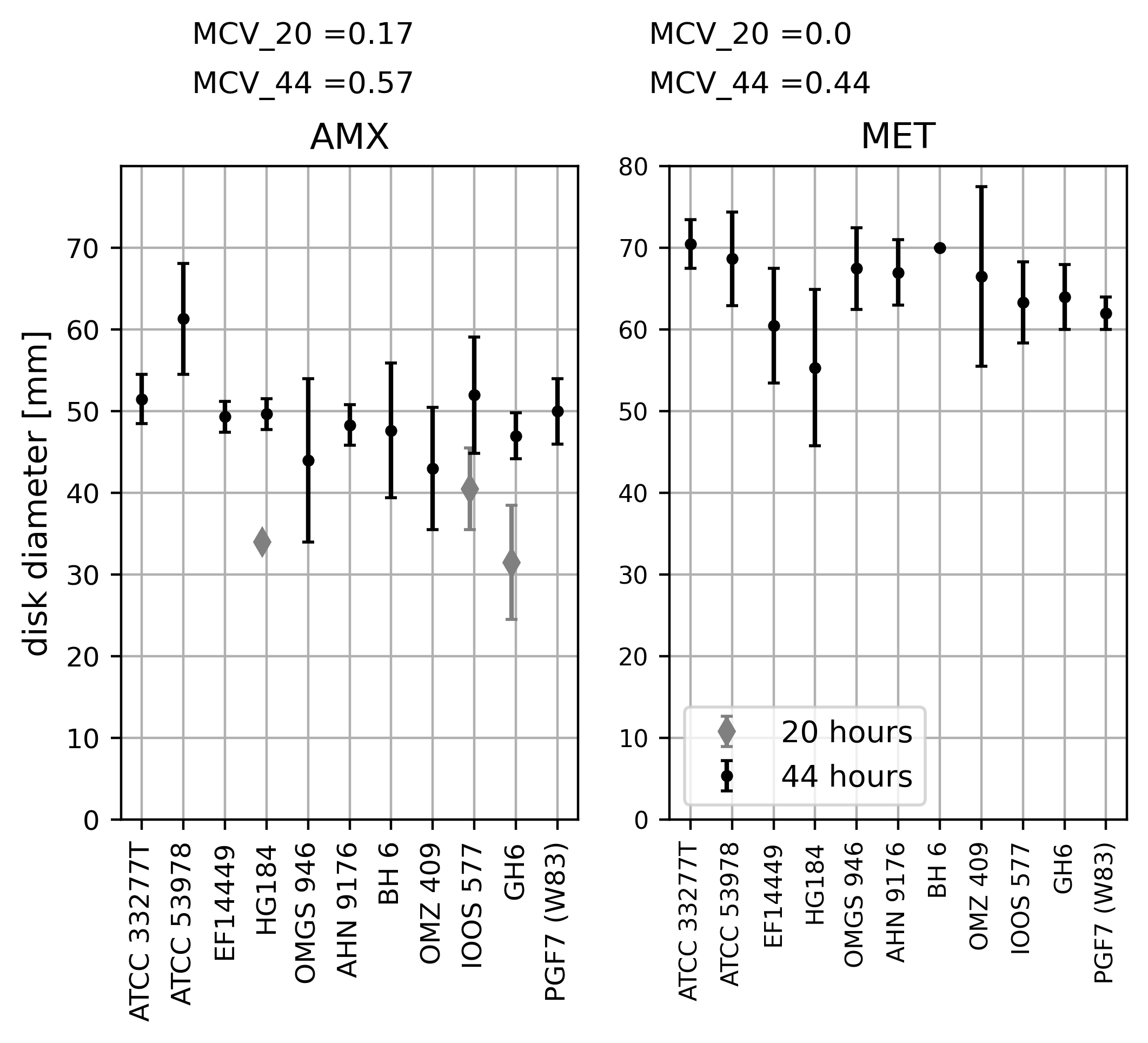

Supplement: Supplementary file 5 — Figure S5. Standard deviation of the inhibition zone diameters coefficient of variance of the mean (MCV) of the amoxicillin (AMX) and the metronidazol (MET) disk after 20 and 44 h on P. gingivalis . The isolates are represented on the x‐axis and the inhibition zone diameter (mm) on the y‐axis. [file APM-133-0-s005.png]

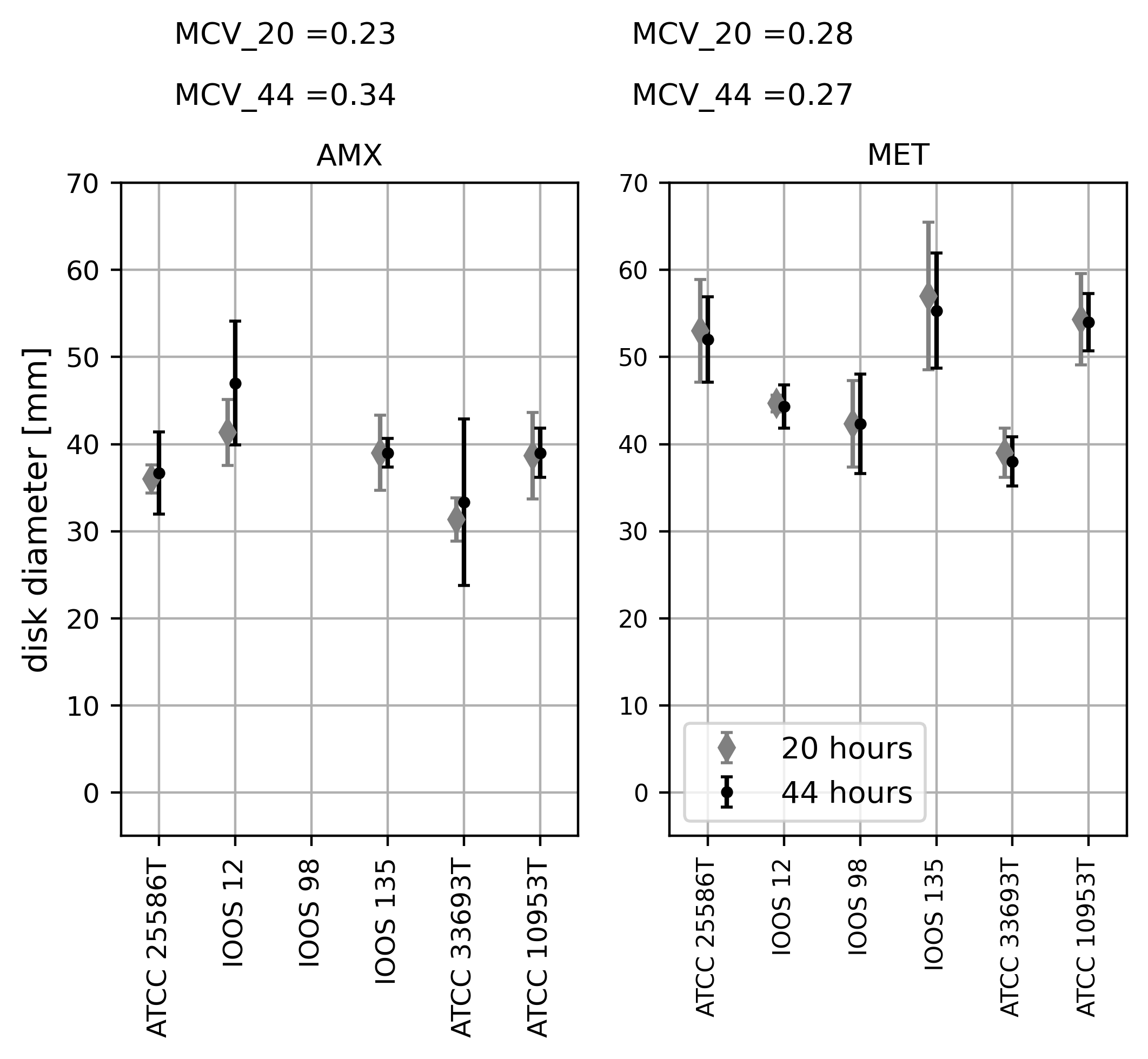

Supplement: Supplementary file 6 — Figure S6. Standard deviation and coefficient of variance of the mean (MCV) of the inhibition zone diameters of the amoxicillin (AMX) and the metronidazol (MET) disk after 20 and 44 h on Fusobacterium. The isolates are represented on the x‐axis and the inhibition zone diameter (mm) on the y‐axis. [file APM-133-0-s006.png]
