# Supplementary material for: The EUCAST Disk Diffusion Method for Antimicrobial Susceptibility Testing of Oral Anaerobes
Source: APMIS. 2025 Feb 9;133(2):e70002. doi: 10.1111/apm.70002 (PMC11807598; doi:10.1111/apm.70002)
Supplement: Supplementary file 7 — Table S1. The number of readings resulting in sufficient growth for interpretation of inhibition zone diameters for 20 Prevotella (60 correlates), 11 Porhyromonas strains (33 correlates), and six Fusobacterium (18 correlates). [file APM-133-0-s004.docx]

**Table S1.** Number of readings resulting in sufficient growth for interpretation of inhibition zone diameters for 20 *Prevotella* (60 correlates), 11 *Porhyromonas* strains (33 correlates), and six *Fusobacterium* (18 correlates).

| Species | AMX | | | | MET | | | | |  |
| --- | --- | --- | --- | --- | --- | --- | --- | --- | --- | --- |
|  | | 20 hours | | 44 hours | | | 20 hours | | 44 hours |  |
| *Prevotella* | 49/60 (82 %) | | 60/60 (100 %) | | | 48/60 (80 %) | | 58/60 (96.7 %) | |  |
| *Porphyromonas* | 11/33 (33.3 %) | | 0/33 (0 %) | | | 31/33 (93.9 %) | | 26/33 (78.8 %) | |  |
| *Fusobacterium* | | 18/18 (100 %) | | 18/18 (100 %) | | | 18/18 (100 %) | | 18/18 (100 %) | |
